# Quality of life in people with transsexuality after surgery: a systematic review and meta-analysis

**DOI:** 10.1186/s12955-020-01510-0

**Published:** 2020-08-03

**Authors:** Mehrdad Eftekhar Ardebili, Leila Janani, Zaher Khazaei, Yousef Moradi, Hamid Reza Baradaran

**Affiliations:** 1 Mental Health Research Center, Psychosocial Health Research Institute (PHRI), Iran University of Medical Science, Tehran, Iran; 2 Preventive Medicine and Public Health Research Center, Psychosocial Health Research Institute (PHRI), Iran University of Medical Sciences, Tehran, Iran; 3 Department of Public Health, School of Medicine, Dezful University of Medical Sciences, Dezful, Iran; 4 Social Determinants of Health Research Center, Research Institute for Health Development, Kurdistan University of Medical Sciences, Sanandaj, Iran; 5 Endocrine Research Center, Institute of Endocrinology and Metabolism, Iran University of Medical Sciences (IUMS), Tehran, Iran; 6 Ageing Clinical & Experimental Research Team, Institute of Applied Health Sciences, Aberdeen, Scotland AB25 2ZD UK

**Keywords:** Male to female, Female to male, Quality of life, WHOQoL-BREF, SF36, Transsexual individuals

## Abstract

**Background:**

Several reports have demonstrated varying results on the quality of life (QoL) of the transgender population. Therefore, the aim of this study was to conduct a systematic review and meta-analysis about the quality of life (QoL) of individuals during the post transsexual surgery period.

**Methods:**

We searched major biomedical electronic databases, including Scopus, Google Scholar, Psychological Information Database (PsycInfo), Web of Science, PubMed, Excerpta Medica dataBASE (EMBASE), and ProQuest, for all relevant literature published in English up to December 2019. The included papers required to be cross sectional studies that reported quality of life in people with transsexuality post surgery. After selecting eligible studies, 2 authors extracted data of each study independently and resolved any inconsistency by consensus with the third reviewer. The risk of bias was assessed by 2 independent research experts by the Newcastle-Ottawa Scale (NOS).

**Results:**

In this study, out of 497 articles extracted from the initial investigation, 8 articles with 1099 patients were ultimately selected for meta-analysis. The pooled mean of quality of life in transsexual individuals was obtained to be 70.45 (95%CI 55.87–85.03) and 59.17 (95%CI 48.59–69.74), based on World Health Organization Quality of Life (WHOQoL-BREF) and The 36-item short form of the Medical Outcomes Study questionnaire (SF36), respectively. Also, the results of the subgroup analysis for the weighted mean quality of life in male to female and female to male showed that the mean quality of life in female to male was 57.54 (95%CI 42.24–72.84) and it was 62.47 (95%CI 45.94–79.00) in male to female, based on SF36 questionnaire. Moreover, the weighted mean quality of life in female to male was 69.99 (95%CI 43.76, 96.23) and it was 70.65 (95%CI 53.11, 88.19) in male to female, based on WHOQoL-BREF questionnaire.

**Conclusion:**

The results of this systematic review may support the approaches to transsexuality that facilitates sex reassignment. In this review, the means of quality of life after surgery were not compared to the means of quality of life before surgery or even before hormonal therapy which was due to inadequate number of primary studies.

## Introduction

Every individual has a significant part of identity called gender identity. It is also one of the most important aspects of human identity including a sense of self and a self-image that every person has it as a man or woman [1]. Whether a human is called a woman or a man is the most important means of identifying and valuing any individual by him/herself and the environment around him/her, which encourages him to strengthen his/her gender-specific behaviors. However, this natural process, which is successful in most cases, may go through a different path in some cases, so that a child doubts about belonging to one of the 2 sexes or completely associate him/herself with the opposite sex. In this situation, s/he suffers from a sexual identity disorder, which is called transsexuality [2].

To date, no clear explanation has been provided for the etiology of transsexuality, although some speculations have been made by physicians, psychiatrists, and biologists. In this regard, the role of biological and psychological factors, such as inheritance, prenatal stress, parental sexual relations, genetic disorders, hormone structure, neurological and central nervous system problems, and some environmental factors, should be taken into consideration [3, 4]. The prevalence of transsexuality is more common among men, with reports of 1 per 30,000, but it is 1 per 100,000 in women. According to the reported statistics, the prevalence of transsexuality is 6 per 100,000 people worldwide [5, 6].

Although the phenomenon of transsexuality seems to be a personal matter, it has consequences in terms of its formation aspects and sociocultural dimensions [7]. Even though the number of people with transsexuality is low compared to those suffering from other diseases, but it can convert to a source of identity crisis due to the transsexuality effects on the personality and behavioral system of the individuals as well as their social adjustment [8, 9].

Transsexuality changes the individual’s life path in the following ways: causing many problems in physical, psychological, social, economic, and family aspects; increasing the feeling of dependence, depression, and isolation; decreasing self-confidence and social capital; and increasing the sense of vulnerability in the patients, which leads to disturbances in daily functions, social activities, and peace of mind. Moreover, transsexuality causes the patients to depend on others and be unable to participate in common social activities. All of these problems, along with various treatments, complications, and high costs of treatments, reduce the quality of life transsexual individuals [10–12]. Defining quality of life is difficult, as it is a broad and complex concept which is recognized by the feeling of satisfaction and happiness. The subjective nature of quality of life addresses people’s own perceptions of their lifestyles rather than reports by others [13, 14]. Therefore, transsexual individuals with similar problems may have different opinions about their quality of life and report it differently [14]. Various studies have been done on the quality of life of transsexual individuals [15–18]. Changing gender is a complex phenomenon that remarkably affects the individuals’ health and social performance and also their identities [19]. It also changes the circle of the individuals’ social roles. Due to their special conditions, people who undergo gender reassignment have less successful communications with different individuals and social groups than ordinary people. Therefore, their social capital is lower than that of ordinary people, so that they sometimes face limitations in meeting their basic needs, such as education, job seeking, marriage, housing, and the need for safety, affection, and communication with others [7].

Transsexuality may affect various dimensions of the individuals’ health in their personal and social lives. Exclusion and isolation from family, friends and relatives, homelessness, and poverty are among the problems that transsexual individuals often encounter, which can reduce their quality of life [19, 20]. Various studies reported that undergoing surgeries for gender change may improve quality of life in several areas, among which is the quality of their social life [21–23]. Therapeutic hormones and surgical procedures can harmonize the biological sex and the identity of the individuals, and thus improving their satisfaction and self-confidence [8, 16, 24]. Based on a recent report by Nobili et al., quality of life in transgender people was lower than the general population, but in their review, the authors measured quality of life (QoL) based on all types of questionnaires in general [25]. All tools and instruments can assess the impact of the disease, but cannot measure the quality of life per se, which has been aptly described as “the missing measurement in health”. WHO (The World Health Organization) has developed and designed The World Health Organization Quality of Life Brief Version (WHOQOL-BREF), which is a measure of quality of life based on a subjective, generic, and cross-cultural evaluation. This scale with The 36-item short form of the Medical Outcomes Study questionnaire (SF36) is a specific tool for measuring quality of life (QoL) and can help researchers to provide a measure of the impact of disease and quality of life [26]. Although the number of people with sexual dissatisfaction is lower compared to patients suffering from other illnesses, its impact on the cognitive and behavioral systems of individuals and on their quality of life is highly important and may become a source of identity crisis. Considering surgery for this group as one the treatment modality regarding that there are several reports with varied results about the impact of surgery on quality of life (QoL) in this group, this study was conducted to perform a systematic review and meta-analysis to measure the quality of life of individuals post transsexual surgery.

## Methods

This systematic review was reported according to the Meta-Analyses of Observational Studies in Epidemiology (MOOSE) [27].

### Search strategy and time period

We searched all relevant literature published up to December 2019 in major biomedical electronic databases, including Scopus, Google Scholar, Psychological Information Database (PsycInfo), Web of Science, PubMed, Excerpta Medica dataBASE (EMBASE), and ProQuest. Researchers performed a search of these databases, with hand searching through the reference lists and grey literature. The key search terms were as follow: **“Transsexual Individual”, “Transsexual Individuals”, “Transsexualism”, “Female to Male (FTM)”, “Male to Female (MTF)”,** “**Gender Dysphonia”, “Gender Incongruence”, “Gender Identity Disorder”, “Quality of Life”, “QoL”,** “**Health-Related Quality of Life”, “Surgery”,** “**Operative Therapy”, “Operative Procedures”, and “Invasive Procedures”**. The final phrases for the search included these terms and their synonyms/various forms linked with appropriate hyphens and as sensitive as possible for any relevant article, according to the instructions of the database of interest. Moreover, we manually reviewed the references of the most relevant articles for any potential study that might have been initially missed while searching the electronic information sources.

**Fig. 1:**
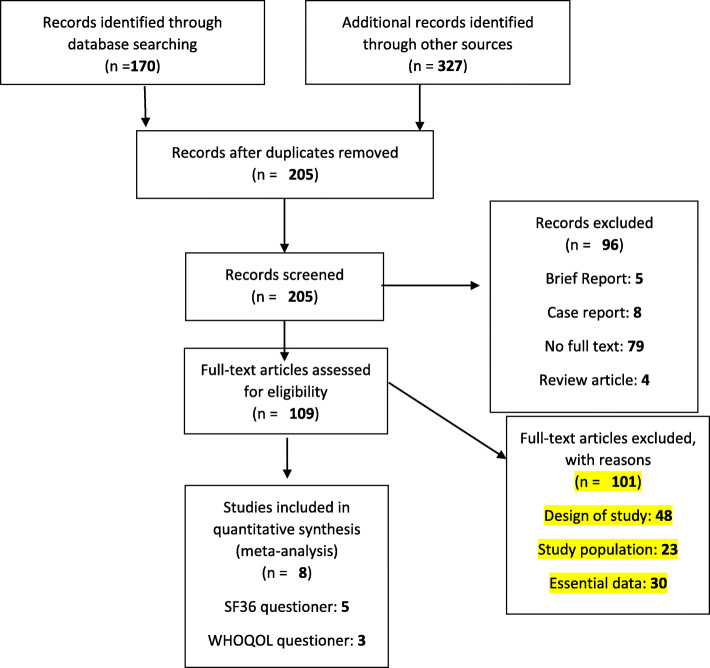
Flow diagram of the literature search and study selection

We exported the search outputs into the End-Note software version 8 and deleted duplicate studies. Two independent researchers (MY and BH) reviewed the primary search results based on inclusion and exclusion criteria and eliminated some of the articles after reviewing the titles and an abstracts. Then, we investigated the search results and excluded some studies after conducting full-text review (Fig. 1). In case of any disagreements about the inclusion/exclusion criteria and data extraction, the third reviewer (EM) assessed the articles for inclusion in the meta-analysis.

### Inclusion and exclusion criteria

After initial screening, all manuscripts were evaluated thoroughly by reaching and reading their full-texts. Inclusion criteria were as follow:

I. Study Design: The included studies required to be cross sectional.
II. Participants: The included study populations were transsexual individuals, those with gender dysphoria and transsexualism, and those with previous diagnoses according to International Classification of Diseases (ICD) or Diagnostic and Statistical Manual of Mental Disorders (DSM) or self-defined as a transgender.
III. Language: The main articles were required to be in English.
IV. Assessment Method: Studies were eligible for inclusion if they had used SF36 and WHOQoL-BREF questionnaires to evaluate quality of life in individuals post transsexual surgery. The SF-36 consists of 36 questions grouped into 8 domains: physical function (domain 1), social function (domain 2), role physical (domain 3), role emotional (domain 4), mental health (domain 5), vitality (domain 6), body pain (domain 7), and general health (domain 8). A score ranging from 0, indicating the worst health status, to 100, indicating the best health status, is assigned for each domain [28]. The WHOQOL-BREF, a 26-item instrument, measures the following broad domains: physical health, psychological health, social relationships, and environment [29, 30]. It contains 2 items on “overall QoL and general health and 24 items on satisfaction, which are evaluated in 4 domains: physical health, (7 items), psychological health (6 items), social relationships (3 items), and environmental health (8 items). Each item is scored on a 5-point Likert scale from 1 to 5, scored in a positive direction, with lower scores denoting a lower QoL, and transformed to a 4–20 score [31].
V. Summary Measures: The selected articles required to report at least the main outcome measurements of interest according to our research questions: quality of life in patients after surgery.

Other study types, including clinical trials, letters, cohorts, case controls, case series, and case reports were excluded. Also, articles that did not report quality of life by WHOQoL-BREF or SF36 were excluded from analysis.

### Data extraction

After eligible manuscripts were selected, 2 independent reviewers (MY and JL) extracted data inconsistencies and corrected and resolved them by consensus and consultation with the third reviewer (BH). Moreover, after sorting out the list of included studies, we performed cross-checking by the first author’s name and year of publication to consider any possibility of data overlap. During the entire data collection, any discrepancy in the procedures was resolved by further assessments and discussions with the third researcher.

### Risk of Bias

Two research experts (MY and KHZ) assessed the quality assessment of eligible remained papers independently by the Newcastle-Ottawa Scale (NOS) [32, 33]. This scale has been adapted from the Newcastle-Ottawa Quality Assessment Scale for cohort and case-control studies to perform quality assessments on cross sectional studies for the systematic reviews. This scale is a modified version of the NOS scale and has been used by several other researchers who felt the need to adapt the NOS scale to appropriately assess the quality of cross sectional studies. We did a comprehensive search of the literature and found that a study with a NOS score of 7 or more can be considered a good study [34]. In addition, quality of included studies assessed by general information about sample representativeness, study participants, sample size, study participants and setting, data analysis, measurement, and confounding factors/subgroups. In this study, Cohen’s kappa statistic was used to determine the agreement between the results of quality assessment of the 2 experts, which was found to be 0.92.

### Statistical analysis

After data extraction, STATA version 14.0 (Stata Corp. College Station, TX, USA) was used for meta-analysis [35, 36]. We extracted the mean and standard deviations (SD) of included articles and pooled them in the meta-analysis. According to the guidelines of WHOQoL-BREF, the raw domain scores for the WHOQOL-BREF were transformed to a score between four and 20. The scores of each domain are scaled in a positive direction (i.e., lower scores denote lower quality of life). The mean score of the items in each domain is used to calculate the domain scores, which are ultimately transformed linearly to a scale of zero (worst measured health) to 100 (best measured health) [37–39]. The SF-36 Health Survey contains 36 items that are scored out of eight scales: physical functioning, role limitations due to physical health problems, bodily pain, general health, vitality, social functioning, role limitations due to emotional problems and mental health. It also includes a single item that provides an indication of perceived change in health. For each scale, a score ranging from zero (worst measured health) to 100 (best measured health) was calculated [40, 41].

A Cochran Q test was conducted to assess heterogeneity and an I^2^ statistic was calculated to estimate the percentage of total variation resulting from between-study variation (31). Low, moderate, or high degrees of heterogeneity were approximated by *I*^2^ values of 25, 50, and 75%, respectively. If the *I*^2^ value was larger than 50%, random-effect model was estimated. Heterogeneity was assessed by subgrouping MTF and FTM, age, domains of questionnaire, type of questionnaire, and country. Publication bias was assessed by Egger and Begg’s test, with a significance level set at *p*-value < 0.10. In addition, funnel plots were planned if more than 10 studies were encountered for each forest plot; however, the number of studies was not found to be adequate for such plotting.

## Results

In this study, out of 497 articles extracted from the initial investigation, 8 [15–17, 24, 42–45] with 1099 patients were ultimately selected for meta-analysis (Fig. 1, Tables 1 and 2). The results of risk of bias assessment are presented in Table 3.

**Table 1:** The characteristics of the SF36 questioner studies included in the analysis

Authors	Year	Type of study	SS^&^	Surgery		QOL* (Mean ± SD)									NOS** Score
				FTM^a^	MTF^b^	D1^c^	D2 ^d^	D3 ^e^	D4 ^f^	D5 ^g^	D6^h^	D7 ^i^	D8 ^j^	Total QOL	
Gorin-Lazard, A. et al. [44]	2012	Cross sectional	31	*		89.4 ± 15.7	76.3 ± 27.3	76.7 ± 34.7	81.1 ± 31.2	70.0 ± 17.3	62.7 ± 16.5	75.9 ± 20.1	79.6 ± 14.5	78.15 ± 18.7	7
Gorin-Lazard, A. et al. [44]	2012	Cross sectional	30		*	92.7 ± 9.0	82.7 ± 20.6	84.7 ± 25.6	77.4 ± 33.8	73.0 ± 17.7	63.4 ± 19.0	74.0 ± 18.1	77.1 ± 17.5	75.55 ± 18.55	7
Newfield, E. et al. [16]	2006	Cross sectional	376	*		51.79 ± 7.6	43.14 ± 10.9	50.59 ± 8.8	42.42 ± 11.6	42.12 ± 10.2	46.22 ± 9.9	49.73 ± 9.9	47.76 ± 10.5	46.99 ± 10.05	7
Vasegh Rahimparvar, F. et al. [17]	2013	Cross sectional	46	*		81.9 ± 16.1	70.11 ± 27.3	65.22 ± 37.1	64.49 ± 38.7	67.22 ± 16.2	63.91 ± 17.7	80.65 ± 23.8	67.17 ± 22.4	66.19 ± 23.10	6
Motmans, J. et al. [15]	2012	Cross sectional	49		*	83.78 ± 18.58	80.10 ± 23.37	76.53 ± 38.33	82.31 ± 34.09	71.51 ± 16.40	60.61 ± 18.16	76.43 ± 22.55	65.51 ± 21.71	76.48 ± 22.10	6
Motmans, J. et al. [15]	2012	Cross sectional	54	*		78.70 ± 25.94	79.63 ± 19.62	72.69 ± 40.71	75.31 ± 36.74	69.26 ± 20.78	60.93 ± 20.58	78.42 ± 28.42	67.50 ± 21.50	74.00 ± 23.72	6
Wierckx, K. et al. [24]	2011	Cross sectional	47		*	85.9 ± 15.0	85.5 ± 19.5	83.3 ± 33.2	83.0 ± 34.1	72.6 ± 19.2	62.1 ± 20.7	75.8 ± 20.8	70.9 ± 19.4	79.40 ± 20.1	6

^a^*MTF* Male to Female, ^b^*FTM* Female to Male; ^c^ domain 1(Physical); ^d^ domain 2(Social); ^e^ domain 3(Role physical); ^f^ domain 4(Role emotional); ^g^ domain 5(Mental health); ^h^ domain 6(Vitality); ^I^ domain 7(Body pain); ^j^ domain 8(General health)

^&^*SS* Sample Size, *QOL Quality of Life, **Newcastle-Ottawa Scale score

**Table 2:** The characteristics of the WHOQOL questioner studies included in the analysis

Authors	Year	Type of study	SS^&^	Surgery		QOL* (Mean ± SD)					NOS** Score
				FTM^a^	MTF^b^	D1^c^	D2^d^	D3^e^	D4^f^	Total QOL	
Thompson, HM. et al. [45]	2015	Cross sectional	312	*		66.08 ± 18.98	67.39 ± 17.84	65.0 ± 22.85	59.54 ± 17.74	64.50 ± 19.35	7
George, A. et al. [43]	2015	Cross sectional	60		*	69.70 ± 17.47	64.86 ± 18.51	66.53 ± 11.73	65.00 ± 13.84	66.52 ± 15.39	6
Başar, K. et al. [42]	2016	Cross sectional	72		*	75.00 ± 0.00	56.00 ± 0.00	97.10 ± 0.00	63.00 ± 44.00	72.77 ± 11.00	6
Başar, K. et al. [42]	2016	Cross sectional	22	*		81.00 ± 0.00	69.00 ± 58.90	75.00 ± 19.00	75.00 ± 0.00	75.00 ± 19.50	6

^a^*MTF* Male to Female, ^b^*FTM* Female to Male; ^c^ domain 1(Physical); ^d^ domain 2(Psychological); ^e^ domain 3(Social); ^f^ domain 4(Environmental)

^&^*SS* Sample Size, *QOL: Quality of Life; ** Newcastle-Ottawa Scale score

**Table 3:** Quality assessment of included studies

Study	Total score	Item 1	Item 2	Item 3	Item 4	Item 5	Item 6	Item 7	Item 8	Item 9	Item 10
Gorin-Lazard, A. et al. [44]	7	Y	Y	Y	Y	Y	Y	NA	Y	NA	NA
Vasegh Rahimparvar, F. et al. [17]	6	Y	N	Y	Y	Y	UC	UC	Y	Y	NA
Newfield, E. et al. [16]	7	NA	Y	Y	Y	Y	Y	Y	NA	Y	N
Motmans, J. et al. [15]	6	NA	UC	Y	Y	Y	Y	UC	Y	Y	NA
Wierckx, K. et al. [24]	6	Y	UC	Y	Y	Y	N	N	Y	Y	N
Thompson, HM. et al. [45]	7	Y	N	Y	Y	Y	NA	Y	Y	Y	NA
George, A. et al. [43]	6	Y	Y	Y	Y	Y	N	N	Y	NA	UC
Başar, K. et al. [42]	6	Y	Y	Y	Y	Y	NA	UC	Y	N	UC

Item _1_: Was the sample representative of the target population?

Item _2_: Were study participants recruited an appropriate way?

Item _3_: Was the sample size adequate?

Item _4_: Where the study subjects and setting described in detail?

Item _5_: Was the data analysis conducted with sufficient coverage of the identified sample?

Item _6_: Were objective, standard criteria used for measurement of the condition?

Item _7_: Was the condition measured reliably?

Item _8_: Was there appropriate statistical analysis?

Item _9_: Are all important confounding factors/subgroups/different identified and accounted for?

Item _10_: Were subpopulations identified using objective criteria?

*Y* Yes, *N* No, *UC* Unclear, *NA* Not applicable

The pooled mean of quality of life after surgery in 633 transsexual individuals was obtained to be 59.17 (95%CI 48.59–69.74) by SF36 questionnaire (Table 4). Also, the results of subgroup analysis for the weighted mean quality of life by MTF and FTM showed that the mean quality of life in FTM was 57.54 (95%CI 42.24–72.84) and it was 62.47 (95%CI 45.94–79.00) in MTF, with 507 and 126 transsexual patients, respectively (Table 4). Moreover, the physical domain had a higher weighted mean [74.53 (95%CI 59.13, 89.92)]. Patients (*N* = 407) aged ≤35 had higher weighted means than patients aged > 35 (*N* = 226) [60.00 (95%CI 44.36, 75.64) vs 60.21 (95%CI 43.88, 76.54)] (Table 4). The weighted mean of quality of life was higher in French transsexuals (*N* = 164) than American transsexuals (*N* = 376) [76.17 (95%CI 53.60, 88.73) vs 48.05 (95%CI 34.33, 61.76)] (Table 4).

**Table 4:** The weighted mean (95% confidence intervals (CIs)) for quality of life by SF36

Subgroups	Number of studies (Sample Size)	Weighted Mean (95% CI)	Range		Between subgroups		Between groups	
					I^2c^ (%)	P _heterogeneity_	Q^d^	P _heterogeneity_
			Minimum	Maximum				
Trans								0.000
MTF^a^	3 (126)	62.47 (45.94, 79.00)	29.94	95.87	12.9	0.328	7.41	
FTM^b^	4 (507)	57.54 (42.24, 72.84)	27.29	87.86	0.0	0.401		
Domains							9.49	0.000
Physical	5 (633)	74.53 (59.13, 89.92)	49.00	92.70	65.9	0.004		
Social	5 (633)	59.57 (38.39, 80.75)	14.00	85.50	66.1	0.004		
Role physical	5 (633)	55.07 (43.52, 66.62)	49.10	84.70	0.0	0.817		
Role emotional	5 (633)	53.30 (40.22, 66.38)	42.42	83.00	0.0	0.746		
Mental health	5 (633)	57.88 (47.91, 67.84)	42.12	73.00	0.0	0.502		
Vitality	5 (633)	54.23 (44.27, 64.18)	46.22	63.91	0.0	0.951		
Body pain	5 (633)	59.62 (48.97, 70.27)	49.10	80.65	0.0	0.555		
General health	5 (633)	59.84 (49.63, 70.04)	47.76	77.10	0.0	0.544		
Mean age							7.52	0.000
≤ 35	2 (407)	60.00 (44.36, 75.64)	49.05	79.40	11.0	0.338		
> 35	3 (226)	60.21 (43.88, 76.54)	46.99	78.15	3.0	0.378		
Geographical eras							5.25	0.004
USA	1 (376)	48.05 (34.33, 61.76)	–	–	–	–		
France	2 (164)	76.17 (53.60, 88.73)	74.00	78.15	0.0	0.990		
Belgium	1 (47)	79.40 (33.15, 87.43)	–	–	–	–		
Iran	1 (46)	66.19 (20.92, 89.78)	–	–	–	–		
Overall	5 (633)	59.17 (48.59, 69.74)	46.99	79.40	0.0	0.487	10.97	0.0001

^a^*MTF* Male to Female, ^b^*FTM*: Female to Male, ^c^*I*^*2*^ I Square, ^d^*Q* Cochran’s Q test

The pooled mean of quality of life in transsexual individuals was obtained to be 70.45 (95%CI 55.87–85.03), with 466 transsexual patients, by WHOQoL-BREF questionnaire (Table 5). Furthermore, the results of subgroup analysis for the weighted mean quality of life by MTF and FTM showed that the mean quality of life in FTM was 69.99 (95%CI 43.76, 96.23) and it was 70.65 (95%CI 53.11, 88.19) in MTF, with 334 and 132 transsexual patients, respectively (Table 5). Also, the social domain had a higher weighted mean of quality of life [68.25 (95%CI 50.32, 86.17)]. Patients aged ≤35 (*N* = 194) had higher weighted means than patients aged > 35 (*N* = 372) [73.31 (95%CI 54.53, 92.09) vs 66.12 (95%CI 42.46, 99.63)] (Table 5).

**Table 5:** The weighted mean (95% confidence intervals (CIs)) for quality of life by WHOQOL

Subgroups	Number of studies (Sample Size)	Weighted Mean (95% CI)	Range		Between subgroups		Between groups	
					I^2c^ (%)	P _heterogeneity_	Q^d^	P _heterogeneity_
			Minimum	Maximum				
Trans								0.000
MTF^a^	2 (132)	70.65 (53.11, 88.19)	66.52	72.77	12.9	0.328	7.41	
FTM^b^	2 (334)	69.99 (43.76, 96.23)	65.54	75.00	0.0	0.401		
Domains							7.46	0.000
Physical	4 (466)	68.04 (42.85, 93.23)	66.08	81.00	0.0	0.888		
Psychological	4 (466)	66.30 (41.70, 90.90)	56.00	69.00	0.0	0.990		
Social	4 (466)	68.25 (50.32, 86.17)	65.00	97.10	0.0	0.919		
Environmental	4 (466)	62.94 (42.18, 83.70)	63.00	75.00	0.0	0.971		
Mean age								0.000
≤ 35	1 (94)	73.31 (54.53, 92.09)	–	–	–	–	7.56	
> 35	3 (372)	66.12 (42.46, 99.63)	65.54	66.52	0.0	0.967		
Overall	4 (466)	70.45 (55.87, 85.03)	65.54	75.00	0.0	0.972	9.47	0.0001

^a^*MTF* Male to Female, ^b^*FTM* Female to Male, ^c^*I*^*2*^ I Square, ^d^*Q* Cochran’s Q test

## Discussions

In the present research, 8 studies [15–17, 24, 42–45] were ultimately analyzed to assess the quality of life of transsexual individuals using SF-36 and WHOQoL-BREF questionnaires. The results of this study showed that the mean scores of quality of life were higher in FTMs, based on the SF36 and WHOQOL questionnaires. The results of the study by Parola et al. showed that the quality of social life as well as the quality of sexual life improved after transsexual surgery. Also, female-to-male individuals had better friendly, professional and social lifestyles than male-to-female ones [46]. Transsexual made people more engaging and active in various social activities, and caused them to have stronger social relationships and get out of social isolation. This improvement in social relationships can increase their quality of life [47, 48]. On the other hand, Kuhn et al. showed that patients’ satisfaction was significantly lower compared to that of the control group [10]. Most studies have been performed on quality of life of clinical patients, but not enough attention has been paid to quality of life of transsexual patients. In line with our findings, Wierckx K et al. showed that the mean of quality of life increased after hormone therapy [24]. Moreover, Dhiordan et al. performed a before-after survey on sex reassignment surgery in Brazilian male-to-female transsexual individuals and found that domains II (psychological) and IV (social relationships) of the WHOQoL-BREF were improved after stereotactic radiosurgery (SRS) in patients compared after surgery compared to before surgery [49].

One study found that the quality of life in such areas as public health, role limitation, and physical and personal constraints was lower 15 years after transsexual surgery [10]. In a study by Newfield et al., it was shown that the quality of life was lower in female-to-male bisexual than in male-to-female participants. Female-to-male participants who had received testosterone had a higher quality of life than non-hormone-treated patients [16]. The apparent characteristics of transsexual individuals, including their voice and face, and their friends and family members’ behaviors were effective in their post-surgery communications in the community; limitations and problems were found to be greater for male-to-female transsexual individuals [22]. In the study by Pitts et al., most of the participants assessed their health as good or very good. Bisexual people had a lower health status than normal people in Australia and New Zealand on the SF36 scale. The rate of depression among transsexual individuals was much higher than ordinary people in Australia, and biological men were twice as likely to experience depression compared to biological women [50]. As transsexualism is an unpredictable phenomenon and the negative attitude of the environment to transsexual individuals may be negative, it causes limitations for the individuals undergoing transsexualism. Limitations such as family and community disapproval may impose the risk of vulnerability to transsexual individuals, which could gradually affect their quality of life and pave the way for their depression [51].

In their study, Rezaei et al. showed that family function, emotional fusion, behavior control, and emotional responsiveness can play a significant role in helping transsexual individuals to accept their new sexual role [52].

The results of the study by Movahed et al. indicated that the mean gender identity disorder among transsexual individuals was much lower than those who did not undergo this type of surgery. A comparison between gender identity disorder in transsexual individuals before and after surgery showed that their disorder was severely reduced after surgery. In addition, the mean total index of the quality of life and level of psychological well-being was different among transsexual individuals compared to nontranssexual individuals; for example, transsexuals had a higher quality of life and mental health compared to nontranssexual individuals [53].

Rakic et al. found that hormone therapy reduced depression and identity disorder in transsexual individuals and increased their quality of life. Although surgery and hormone therapy slightly improved the quality of life of transsexual individuals, their widespread social and interpersonal problems were much more than nontranssexual individuals [48].

In a study by Rahimparvar et al., the quality of life of transsexual women was almost the same as that of nontranssexual women (Quality of life in both groups were moderate.). However, the mean total score of quality of life in transsexual women was slightly higher than that of nontranssexual women [17]. In a study by Weyers et al., the mean total score of quality of life was 73.3 ± 25.11 in transsexual women [18]. One study showed that the life quality score depended on gender, and the mean score of quality of life in women was lower than that of men (35). TS person who still has a girl’s body, even though wearing males’ clothes and having males’ behaviors, may be seen as a strong and daring girl and such masculine appearance and behavior may be perceived as a sign of reliability [54].

However, if MTF person has women-specific behaviors, he will be severely excluded from the community and will be considered a weak and womanly person. After transsexual surgeries, MTF individuals will formally and legally be in the position of women and will quite irreversibly face discrimination and limitations of women, which may gradually reduce their quality of life [54].

The results of prior studies showed that the mean score of quality of life in transsexual people decreased with age. Because when the age, physical problems and physical pain increase, their quality of life increase too [55, 56].

The results of different studies showed that marital status affected the mean score of quality of life. Due to social and economic pressures, divorced women felt helpless and, despite governmental and nongovernmental financial support, they often had limited and painful lives, and their difficult life circumstances made them feel depressed [57, 58]. The results of various studies showed that the mean score of TS people’s quality of life increased with higher levels of education, as educated people usually feel more psychologically secure. Also, the mean quality of life was lower in unemployed transsexual individuals than in others [59].

Results of a review by Nobili et al. showed that transsexual people have poorer mental health QoL compared to the general population. Also, QoL in participants who were exclusively post- cross-sex hormonal treatment (CHT) found no difference in mental health QoL between groups, but in our systematic review, which was based on WHOQoL-BREF questionnaire, it was found that transsexual people have poorer environmental QoL and based on SF36 have poorer role emotional QoL. In addition, the pooled weighted mean of QoL in our review had a lower heterogeneity (I2) than a recent review by Nobili et al., because we included only cross sectional studies and calculated weighted mean based on WHOQOL-BREF and SF36 questionnaire [25].

### Limitation

In this review, the means of quality of life of individuals before transsexual surgery were not compared to their means of quality of life before surgery or even before hormonal therapy, because the number of primary studies was inadequate.

## Conclusion

It seems that the weighted mean of QoL was better in transsexual individuals after surgery, but these results need to enough studies for compare to means of QOL before surgery with after surgery. Transsexuals remain a population at risk for low QoL and mental health. Therefore, it is suggested to pay more attention to different aspects of their treatment, including psychological and physical aspects. The main finding of this study may support the approaches to transsexuality that facilitate sex reassignment.

## Data Availability

The study data extracted for analyses in the current publication are available from the corresponding author upon reasonable request.

## Abbreviations

CI: Confidence Interval
vs: In Compare
FTM: Female to Male
MTF: Male to Female
QoL: Quality of Life
TS: Transsexualism
EMBASE: Excerpta Medica dataBASE
NOS: Newcastle-Ottawa Scale
WHOQOL: World Health Organization Quality of Life
SF36: The 36-item short form of the Medical Outcomes Study questionnaire
WHO: The World Health Organization
WHOQOL-BREF: The World Health Organization Quality of Life Brief Version
MOOSE: The Meta-Analyses of Observational Studies in Epidemiology
PsycInfo: Psychological Information Database
ICD: International Classification of Diseases
DSM: Diagnostic and Statistical Manual of Mental Disorders
SD: Standard Deviations
CHT: Cross-sex Hormonal Treatment
SRS: Stereotactic Radiosurgery
